# Are freely chosen actions generated by stimulus codes or effect codes?

**DOI:** 10.3758/s13414-020-02081-4

**Published:** 2020-07-08

**Authors:** Markus Janczyk, Christoph Naefgen, Wilfried Kunde

**Affiliations:** 1 Department of Psychology, University of Bremen, Bremen, Germany; 2 Faculty of Psychology, FernUniversität Hagen, Hagen, Germany; 3 Department of Psychology III, University of Würzburg, Würzburg, Germany

**Keywords:** Action control, Intention, Action effects, Free choice, Forced choice

## Abstract

A long-standing debate revolves around which mental codes allow humans to control behavior. The internal stimulus model (going back to Rudolf Hermann Lotze) proposes that behavior is controlled by codes of stimuli that had previously preceded corresponding motor activities. The internal effect model (going back to Emil Harleß) proposes that behavior is controlled by codes of perceptual effects that had previously resulted from corresponding motor activities. Here, we present a test of these two control models. We observed evidence for both models with stronger evidence for the internal stimulus model. We suggest that the proposed experimental setup might be a useful tool to study the relative strengths of stimulus control and effect control of behavior in various contexts.

Humans normally have some control over what they do. That is, they have some freedom of how to behave in a given situation and experience themselves as the cause of that behavior. While it is obvious that humans can act in such a self-determined way, it is a fundamental question as to which mental processes enable such control. Starting from the 19th century, two approaches to this question have been proposed, the *internal stimulus model* and the *internal effect model*. Both approaches share assumptions. They both propose that actions are generated by means of codes of perceptual events, which had become linked to motor activities by previous learning experience. They differ, though, in the type of perceptual codes doing so (see Fig. 1).

**Fig. 1:**
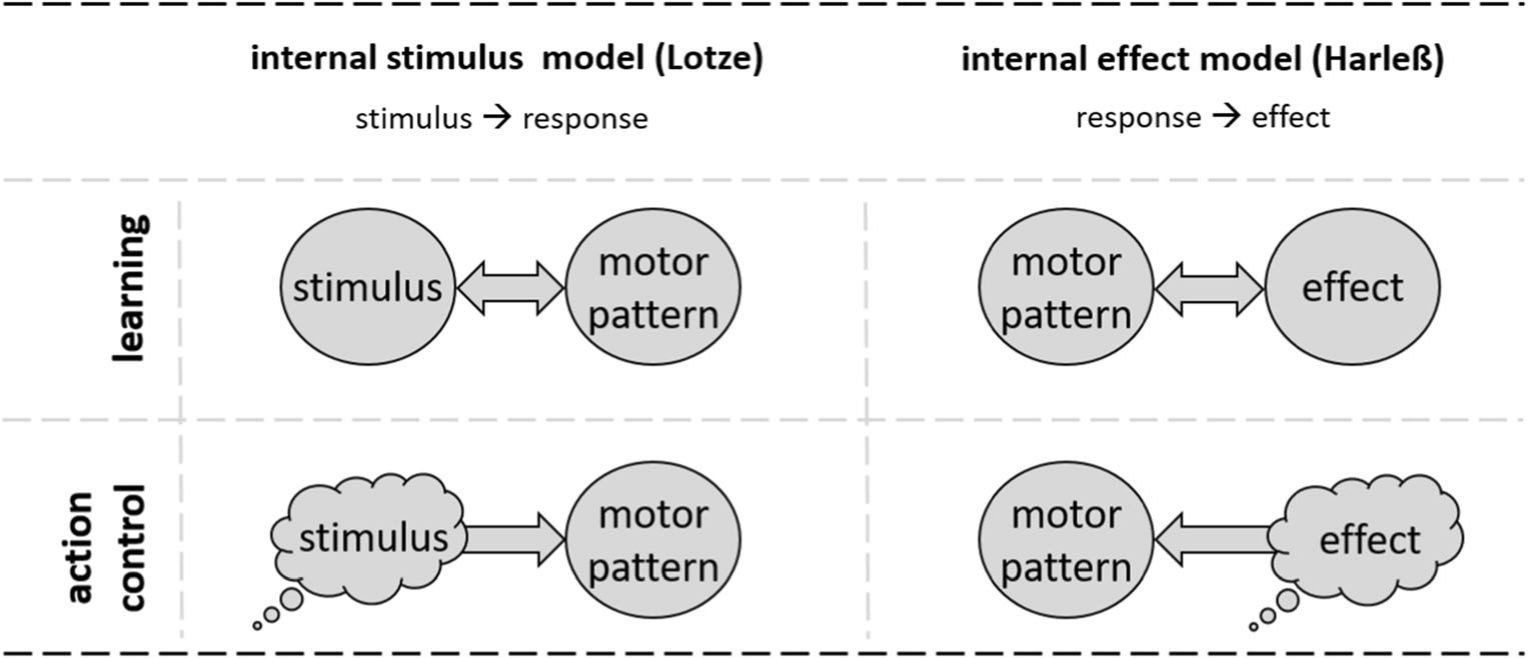
The models of action control according to Lotze and Harleß. According to Lotze’s model (left column), actors first observe themselves responding to certain stimuli. Mental codes of these stimuli then allow intentionally retrieving the corresponding motor patterns. According to Harleß’ model (right column), actors first observe themselves producing certain perceptual effects by motor patterns. Mental codes of these effects then allow intentionally retrieving the corresponding motor pattern (figure adapted from Hommel, Brown, & Nattkemper, 2016)

The internal stimulus model dates back to Rudolf Hermann Lotze (1852). Lotze assumed that humans first acquire stimulus–response links by observing themselves responding to certain situations in specific ways. Later, humans generate behavior intentionally by imagination, rather than actual experience, of corresponding stimulation:

Hier wie überall kann daher der Wille nur jene inneren psychischen Zustände erzeugen, welche der Naturlauf zu Anfangspunkten der Wirkung nach aussen bestimmt hat; die Ausführung der Wirkung dagegen muss er der eigenen unwillkürlichen Kraft überlassen, mit der jene Zustände ihre Folgen herbeizuführen genöthigt sind.^1^ (Lotze, 1852, p. 301)

In other words, humans gain control over behavior via *stimulus* control, that is, by exposing themselves to mentally simulated stimulation that then activates those responses, which had been previously given to such (nonsimulated) stimulation (see Fig. 1, left column). This view has been elaborated by Vygotski (1962) with a focus on verbal stimulation. The idea here is that young humans first carry out certain types of behavior as a response to verbal instruction. Later, they gain control over behavior by (c)overtly imposing self-instructions (inner speech), an idea that has received considerable support in research on control of normal (e.g., Goschke, 2000) and dysfunctional behavior (Meichenbaum, 1977). In sum, control of behavior is achieved by codes of stimuli—that is, perceptual events that had *preceded* previous behavior, which then serve as mental retrieval cues for this behavior.

A different approach has been put forward by Emil Harleß (1861; see Pfister & Janczyk, 2012, for an English translation). In contrast to Lotze, Harleß focused on the stimulation that consistently *follows—*rather than precedes—motor behavior:

Eine intensive Empfindung des Effektbildes ist also allerdings das primäre und unerläßliche Erforderniß für die Ausführung einer willkührlichen Bewegung, aber sie ist nicht das vollkommen Effektuirende dabei.^2^ (Harleß, 1861, p. 67)

In other words, humans first acquire links between bodily movements and the stimulation that consistently follows these movements (i.e., the action *effects*; see Fig. 1, right column). After learning, control over bodily movements is possible by imagination of these effects. That means that humans gain control over their behavior via *effect* control—that is, by exposing themselves to mentally simulated effects, which then activate those motor patterns that are linked to these effects. This view has recently gained considerable support (e.g., Elsner & Hommel, 2001; Janczyk, Durst, & Ulrich, 2017; Janczyk & Kunde, 2020; Janczyk & Lerche, 2019; Koch & Kunde, 2002; Kunde, 2001; for recent reviews, see Badets, Koch, & Philipp, 2016; Shin, Proctor, & Capaldi, 2010). Thus, control over behavior is achieved by codes of effects—that is, perceptual events that had followed previous behavior, which then serve as mental retrieval cues for that behavior. The main structural difference between these two accounts of action control is whether, in the preceding learning events, the stimulation connected with the behavior happens before or after the behavior.

In the present study, we provide a simple experimental test in which the (assumed) lingering codes of internal stimuli and/or internal effects can express themselves independently. We focused on a situation in which the choice of what to do was relatively free to the actor within moderate constraints, because there was neither a specific stimulus to respond to, nor a specific effect to aim at (a free-choice task; Berlyne, 1957). The question we ask is: Is the generation of the eventually executed behavior mediated by (1) codes of stimuli (Lotze’s internal stimulus model) or by (2) codes of effects (Harleß’ internal effect model), which were linked to that behavior, respectively. Thus, we do not ask *why* a specific choice has been made, but *how* this choice is transformed into overt behavior. This is a more basic question than is addressed in theories that are more specific. For example, the generation of a motor activity has been construed as the retrieval of or the specification of parameters of a motor program (e.g., Keele, 1968; Schmidt, 1975). Viewed like this, the present study addresses whether stimulus codes or effect codes serve as retrieval cues or as the basis for specifying such parameters in free choices.

Specifically, we intermixed forced-choice trials and free-choice trials in the experiment (see Fig. 2 for an illustration of the following). In *forced-choice trials*, participants responded to vertically arranged stimuli (square above or below the center of screen) with a nonspatial response (pressing a button once or twice). The responses contingently produced horizontally arranged effects (a square left or right to the center of screen depending on the response). Thus, a particular response led to an apparent movement of the square, and participants are expected to establish links between their behavior and the effects of that behavior.

**Fig. 2:**
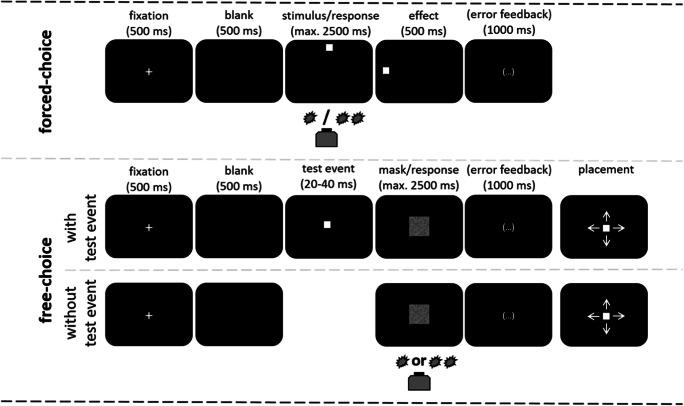
Illustration of trial procedure. Note that forced-choice trials are only illustrated with one particular stimulus and effect, and error feedback was only presented if warranted. Placement was without time limit, and the next trial started after an intertrial interval of 1,000 ms

Two types of free-choice trials were employed. In *free-choice trials with test event*, a masked and thus barely visible square was shown (the *test event*) near the center of the screen. Participants were instructed to choose between responding with one or two key presses, irrespective of whether they saw the rectangle or not. After responding, participants placed a small rectangle where they believed it to have appeared. We expected a rather veridical location report in these free-choice trials that also served to induce participants’ confidence that barely perceptible rectangles would occur, and as a check that they complied with task instructions.

Our main interest relates to another portion of free-choice trials though, where *no* rectangle was actually presented (*free choice without test event*). If the generation of the eventually executed action was mediated by an internally generated code of a certain stimulus, we expected the “perceived” location to resemble, and thus to be biased towards, the vertical position where the stimulus for that action would normally appear. In contrast, if the execution of the eventually chosen action was mediated by a code of a certain effect, we expected the reported location to be biased towards that horizontal position where the effect of that action would normally appear. Such biases towards highly activated perceptual representations due to top-down influences are commonly observed (for a review, see, e.g., Summerfield & Egner, 2009). Because both types of free-choice trial included the presentation of a mask, they are hard to distinguish for participants, who did not know about this distinction, anyway. One could also say that participants responded freely if they could not figure out a clear forced-choice stimulus.

## Method

### Participants

Thirty-two people from the Tübingen area participated (Mean age = 23.7 years, 27 female, five male) for monetary compensation. All participants reported normal or corrected-to-normal vision, were naïve regarding the underlying hypotheses, and provided written informed consent prior to data collection.

### Apparatus and stimuli

Stimulus presentation and response collection happened on a PC connected to a 17-inch CRT monitor (1,024 × 768 resolution). Stimuli were white squares (30 × 30 px) displayed above or below (150 px) the center of the screen (forced-choice trials) or near the center (± 70 px) of the screen (free-choice trials with test event). Action effects were the same white squares displayed left or right (150 px) of the center of the screen. Masks were five images of white noise displayed at the center of the screen. The background was black. The manual responses were given with a response key placed under a finger of the participants. The placement of where participants thought the stimulus appeared in the free-choice trials was controlled by the arrow keys on the keyboard.

### Tasks and procedure

The task was either to give a predefined response (one or two button presses) to the two forced-choice stimuli or to choose freely within moderate constraints one of the two possible responses in free-choice trials (see also Fig. 2). A trial began with the presentation of a central fixation cross (500 ms), followed by a blank screen (500 ms) and the appearance of the stimulus. In forced-choice trials, the stimulus remained visible for 2,500 ms or until a response was given. In free-choice trials with a test event, the test event (the same white square as used as stimuli and effects in forced-choice trials) was visible for a randomized amount of time between 20 and 40 ms. The mask was displayed for 2,500 ms or until a response was given. After the first key press, there was a window of 200 ms to produce a second key press (potentially allowing for second key presses registered that were given up to 200 ms outside of the 2,500 ms window); otherwise, the response was counted as a single key press. After 200 ms or the second key press, the relevant action effect was displayed for 500 ms. General errors (i.e., no response within the time limit) and erroneous responses (wrong number of key presses in forced-choice trials) triggered respective feedback (1,000 ms). After responding in free-choice trials, the participant had an unlimited amount of time to place the rectangle where they thought it appeared before it was masked. The next trial started after an intertrial interval (ITI) of 1,000 ms.

After a mini-block of four trials, during which all trial types occurred, every participant performed six blocks (80 trials each). Within each block, there was an equal number (20 each) of forced-choice trials requiring one response, two responses, free-choice trials with, and free-choice trials without a test event.

An even distribution of responses composed of one and two key presses over the course of the experiment as well as the avoidance of strategies in the free-choice responses were instructed at the beginning of the single individual test sessions of about 60 minutes. When participants responded unevenly (≥80% of responses composed of either one or two key presses), the data were discarded, and the participant was replaced by a new participant to ensure that both response options were at least chosen approximately equally often (*n* = 20 participants). This is a large number of participants, and is larger than in previous studies where we used similar criteria. At present, we can only speculate that the particular choice (1 vs. 2 key presses here; left vs. right key press in most other studies) plays a role in this. The forced-choice stimulus–response mapping and the response–effect mapping were counterbalanced across participants.

### Design and analyses

The dependent variables of most interest were the deviation of the participants’ stimulus placement on the vertical and the horizontal axes in pixels towards the corresponding stimulus/effect. Each free-choice trial was categorized based on whether the response-associated stimulus (in a forced-choice trial) would be located above or below the center and whether the response-associated effect (in a forced-choice trial) would be located left or right from the center. Deviations were then remapped so that positive values indicated a bias toward the stimulus (*y*-axis) or the effect (*x*-axis), and negative values indicated a bias away from the stimulus or the effect.

The central analyses were *t* tests against a mean of zero-pixel deviation for each of those variables for the free-choice trials without a test event. Additionally, a paired *t* test was performed in which the sizes of the deviations from the center towards the corresponding stimulus and towards the corresponding effect were compared with each other. A similar analysis for the free-choice trials with a test event assessed whether the participants’ stimulus placement differed from the actual position of the stimulus. Mean correct response times (RTs) of all trial types were submitted to an ANOVA (forced choice vs. free-choice with test event vs. free-choice without test event). RTs were measured from stimulus/mask onset until the (first) key press.

Supplemental tests were run on free-choice trials with a test event. In these trials, the test event was positioned randomly and always had a deviation from screen center on the *y*-axis and on the *x*-axis. Trials were then classified according to whether the location above/below or left/right of the screen center would fit with one or two responses, respectively, according to the requirements in forced-choice trials. Then, the mean choice rate of congruent response choices was calculated per participant and evaluated with *t* tests against a value of μ_0_ = 0.5.

Trials were excluded from RT analyses as outliers if their RTs deviated more than 2.5 standard deviations from the respective cell mean (calculated separately for each participant; 2.63%). All *p* values were Greenhouse–Geisser adjusted when the assumption of sphericity was violated. In these cases, the respective epsilon is reported.

## Results

### Location biases

**Fig. 3:**
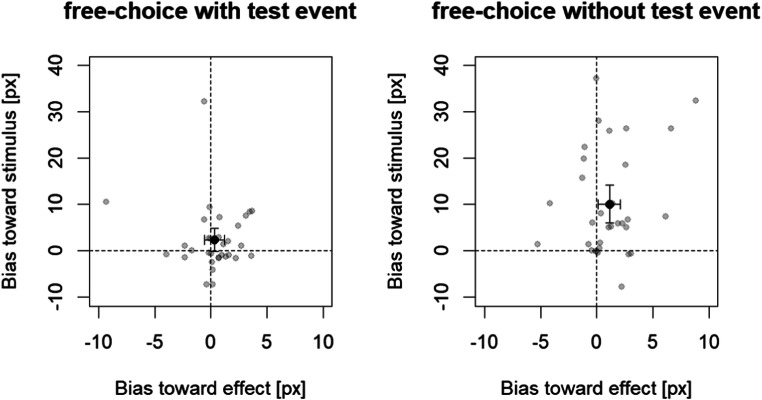
Biases on the *x*-axis and *y*-axis (toward effect and stimulus, respectively) in free-choice trials with a test event (left panel: deviation from the test event center) and without a test event (right panel: deviation from screen center). Positive values indicate biases toward the corresponding stimulus/effect belonging to the chosen response; negative values indicate biases away from this stimulus/effect. Grey circles are individual participants, and the black circle represents the mean with the whiskers indicating 95% confidence intervals

Overall, one key press was given in 58.8% (range: 37.3%–76.2%) of the free-choice trials. In free-choice trials with a test event, the percentage was 53.0 (range: 25.4%–71.4%) and in free-choice trials without a test event, the percentage was 64.9 (range: 25.0%–85.0%). Deviations of the participants’ stimulus placement from the stimulus center (free-choice trials with a test event) and from the screen center (free-choice trials without a test center) are visualized in Fig. 3.

For free-choice trials with a test event (see Fig. 3, left panel), biases were neither significant toward the stimulus (*y*-axis), *t*(31) = 1.92, *p* = .063, *d* = 0.34, nor toward the effect (*x*-axis), *t*(31) = 0.70, *p* = .490, *d* = 0.12, nor did these nonsignificant biases differ significantly from each other, *t*(31) = 1.55, *p* = .132, *d* = 0.27. Most importantly, for free-choice trials without a test event (see Fig. 3, right panel), there was a significant bias toward the stimulus (*y*-axis), *t*(31) = 4.99, *p* < .001, *d* = 0.88, and toward the effect (*x*-axis), *t*(31) = 2.32, *p* = .027, *d* = 0.41. The former bias was larger in size than was the latter bias, *t*(31) = 4.57, *p* < .001, *d* = 0.81.^3^

### Congruent choice rates in free-choice trials with test event

Participants responded congruent with the stimulus position in 68.6% of the trials, *t*(31) = 7.61, *p* < .001, *d* = 1.34. No comparable effect was observed for congruency with effect position (51.2%), *t*(31) = 1.29, *p* = .207, *d* = 0.23.

### RTs and errors

The three task types differed in their average RTs, *F*(2, 62) = 52.63, *p* < .001, η_p_^2^ = .63, ε = .76. Paired *t* tests indicated differences between all three task types, with forced-choice trials (466 ms) having a shorter average RT than both free-choice trials with a test event (726 ms), *t*(31) = 9.27, *p* < .001, *d* = 2.32, and those without a test event (823 ms), *t*(31) = 7.98, *p* < .001, *d* = 1.99. Furthermore, free-choice trials with a test event had shorter RTs than those without a test event, *t*(31) = 2.95, *p* = .006, *d* = 0.74. In the forced-choice trials, the wrong number of key presses was given on average 10.5% of the time, ranging from 2.9% to 22.1%.

## Discussion

Which mental codes trigger freely chosen actions? Codes of stimuli or codes of effects to which the chosen actions are linked? The present study suggests: both. When people generate a motor action, they tend to report perceptual events that resemble both, the stimulus that normally requires the generated actions as well as the effect that normally results from that action. We consider this as strong evidence for the internal activation of corresponding event codes because in the crucial condition, no actual event was presented.

At first glance, it might come to a surprise that we did not observe evidence exclusively for activation of either stimulus *or* effect codes, but rather for both. This might be explained by the assumption that actions are generated neither by stimulus codes nor by effect codes alone, but by codes that represent the transition of a specific stimulus to a subsequent specific effect (cf. Kunde, Schmidts, Wirth, & Herbort, 2017, for some preliminary evidence for this). In general, actions transform the perceptual world prior to acting into another state after acting, as was the case in the present experiment: Responding, for example, to a rectangle at the top by a single key press transformed that rectangle to, for example, a rectangle on the left. If actions were generated by codes of the specific transition they produce, these codes would necessarily resemble the initial state of that transition (i.e., the stimulus) as well as its end state (i.e., the action “effect”).

Admittedly, the bias towards action-contingent stimuli was overall larger than the bias towards action-contingent effects regarding the placements of the test event. In addition, a priming of a particular response in free-choice trials with a test event (akin to that reported by Elsner & Hommel, 2001) was only depending on the test event’s position along the *y*-axis—that is, depending on the stimulus position. This might be because such biases express themselves more strongly when judging stimuli on the vertical rather than horizontal dimension. Alternatively, stimuli might have received more attention in this particular setup, because they were clearly task relevant whereas the ensuing effects were not. The impact of action effects might become larger if they are made task relevant (see Janczyk, Yamaguchi, Proctor, & Pfister, 2015), and in fact, the question of which codes express themselves more strongly under which conditions might be an interesting question, and the present setup might be a useful tool to address such questions. However, this does not undermine the main outcome of the present study—namely, that both of these codes are basically involved in the generation of corresponding motor activities.

One may further argue that our observation in free-choice trials without test event reflects a bias to report corresponding events without actually having a corresponding perception. Nonperceptual explanations for putatively perceptual phenomena are a notorious problem (see, e.g., Firestone & Scholl, 2014). However, two arguments speak against this. First, if there was a bias to report events that resemble stimuli or effects, the same bias should occur when an actual corresponding event was presented. However, the report of actual events was largely unbiased (see Fig. 3, left panel). Second, participants were not informed that occasionally no event occurred. Rather, they were told that they may sometimes see and sometimes not see a square in free-choice trials because of the mask. To us it seems rather unlikely, then, that a response bias would selectively affect some of the free-choice trials only (i.e., those actually without a test event).

We have intermixed forced-choice and free-choice tasks, and this may limit how we can generalize our conclusion to situations where only free-choice tasks are administered. First, the history of experienced forced-choice trials might influence choices, in particular when participants are not entirely free in their choices but need to attain a certain ratio of choices (see Gozli, 2019, for a review and discussion of free-choice tasks and their problems). We cannot exclude that forced-choice trials had an unspecific influence; but certainly, such influence should not affect stimulus or effect imagination selectively and thus not undermine our conclusion. Second, we believe that intermixing both task types reflects human everyday behavior quite well. Frequently, humans switch between situations where the environment strongly suggests a goal (as in the forced-choice trials) and ones where the environment suggests goals less strongly (as in the free-choice trials). We contend that in such situations, and even in situations that clearly fall within the latter category, our conclusion is applicable: we simply do not know which stimulus one imagines in these cases. Our experimental approach, in contrast, created a situation where we had good control over the stimuli and effects participants associated with particular actions. Of course, this comes with some disadvantages as well, but still provides a simplified approximation to the situation under investigation.

In a broader context, it is discussed what free-choice tasks essentially are and whether they do operationalize what they are intended to operationalize. This discussion revolves mostly around the putative distinction of *externally triggered/stimulus-based actions* versus *self-generated/intention-based actions,* which are experimentally addressed by using forced-choice and free-choice tasks (e.g., Brass & Haggard, 2008; Herwig, Prinz, & Waszak, 2007; Waszak et al., 2005). A thorough discussion is beyond the scope of this article (see Gozli, 2019). Briefly, recent work suggested that free-choice tasks are essentially random-generation tasks (Naefgen & Janczyk, 2018) and exhibit mutual priming effects (e.g., Kiesel et al., 2006; Mattler & Palmer, 2012; Naefgen, Caissie, & Janczyk, 2017). Consequently, forced-choice and free-choice tasks are more and more conceived of in a unified framework instead of reflecting two qualitatively different actions (or action systems; see also Bermeitinger & Hackländer, 2018; Naefgen, Dambacher, & Janczyk, 2018; Richardson, Pfister, & Fournier, 2020).

In sum, our experiment revealed that both stimulus codes, and, to a lesser extent, effect codes are indeed imagined when it comes to carrying out a freely chosen action in a free-choice task. Future studies may more thoroughly investigate circumstances changing the relative impact of these codes, and we believe that the present experiment provides a valuable tool to further investigate the importance of imagined stimulus codes and effect codes in action control.

## Footnotes

1 “Here, as everywhere, the will can only generate those internal mental states that the course of nature determined to be the starting points of the outward effect; the execution of these effects, by contrast, must be left up to the involuntary force with which these mental effects are compelled to bring about their outcomes” (translated by the authors).

2 “An intense sensation of the effect image is thus the primary and indispensable prerequisite for the execution of a voluntary movement, but it is not what completely effectuates the movement” (translation from Stock & Stock, 2004, p. 182).

3 When excluding the two participants associated with the outlier values in Fig. 3 (left panel), the qualitative pattern of results remained the same.
